# HLA-B27 is associated with reduced disease activity in axial spondyloarthritis

**DOI:** 10.1038/s41598-021-91829-5

**Published:** 2021-06-10

**Authors:** James T. Rosenbaum, Michael H. Weisman, Hedley Hamilton, Cassie Shafer, Elin Aslanyan, Richard A. Howard, Kimberly Ogle, John D. Reveille, Kevin L. Winthrop, Dongseok Choi

**Affiliations:** 1 Departments of Medicine, Ophthalmology, and Cell Biology, Oregon Health & Science University, Portland, OR USA; 2 Legacy Devers Eye Institute, Portland, OR USA; 3 Cedars Sinai Medical Center, Los Angeles, CA USA; 4 Any-3, London, UK; 5 Spondylitis Association of America, Los Angeles, CA USA; 6 Department of Medicine, University of Texas, Houston, Houston, TX USA; 7 OHSU-PSU School of Public Health and Departments of Medicine and Ophthalmology, Oregon Health & Science University, Portland, OR USA

**Keywords:** Ankylosing spondylitis, Autoimmunity

## Abstract

HLA-B27 is associated with increased susceptibility and disease activity of ankylosing spondylitis, but the effect of HLA-B27 on the activity of the broader category now called axial spondyloarthritis (AxSpA) is apparently the opposite. A modified Bath Ankylosing Spondylitis Disease Activity Index (BASDAI) was used to assess disease activity among 3435 patients with spondyloarthritis (SpA) who participated in a survey designed to assess the effect of their disease and its treatment on the susceptibility and severity of Covid-19. Chi square testing was used to compare BASDAI scores between HLA-B27 positive and negative subjects. 2836 survey respondents were HLA B27 positive. The average BASDAI for the HLA-B27 negative cohort was 4.92 compared to 4.34 for the HLA-B27 positive subjects. Based on linear regression, a subject’s sex could not fully account for the differing BASDAI score in HLA-B27 negative subjects compared to those who are HLA-B27 positive. The difference between B27 positive and negative subjects was skewed by those with a BASDAI score of one or two. HLA-B27 positive subjects were more than twice as likely to have a BASDAI score of 1 compared to HLA B27 negative subjects and about 60% more likely to have a BASDAI score of 2 (p < 0.0001). HLA-B27 positive subjects have less active spondyloarthritis compared to HLA-B27 negative subjects as measured by a BASDAI score. Our data indicate that patients with mild back pain and a diagnosis of AxSpA are disproportionately HLA-B27 positive. The HLA-B27 test facilitates the diagnosis of axial spondyloarthritis such that patients from a community survey with mild back pain may be disproportionately diagnosed as having AxSpA if they are HLA-B27 positive. The test result likely introduces a cognitive bias into medical decision making and could explain our observations.

## Introduction

The major histocompatibility complex allele, HLA-B27, is a risk factor for the development of ankylosing spondylitis (AS)^1,2^. Its presence is widely reported to correlate with susceptibility as well as the activity of this disease^3–9^, with activity measured by a Bath Ankylosing Spondylitis Disease Activity Index (BASDAI). A BASDAI is a widely accepted measure for disease activity^3–9^. It is based on patient responses to 6 questions. It uses a Likert scale and averages the responses.

The New York criteria provide classification criteria for ankylosing spondylitis and require definite radiographic evidence for sacroiliitis on a plain x-ray^10^. The realization that sacroiliac inflammation occurs in advance of and does not always result in radiographic change led to the ASAS (Assessment of Spondyloarthritis) criteria for the classification of axial spondyloarthritis (AxSpA). These criteria have had a major impact on the diagnosis and understanding of axial spondyloarthritis^11^. The recognition of non-radiographic (nr) AxSpA is an advance that is benefiting many patients who were previously not diagnosable^12^. Thus, spondyloarthritis is an umbrella term that includes both ankylosing spondylitis and nr-AxSpA. It is also sometimes used to describe individuals with only peripheral joint disease that does not affect the spine^13^. Everyone with ankylosing spondylitis should meet criteria for the diagnosis of AxSpA, but many with AxSpA do not meet criteria for ankylosing spondylitis. AxSpA differs from AS in several major respects. For example, in contrast to AS, AxSpA is more common in females^14,15^. Despite the absence of radiographic changes in the sacroiliac joints of many patients with AxSpA, the disease activity in AxSpA is generally comparable to the disease activity in AS as judged by the BASDAI^14,15^.

The effect of HLA-B27 on disease activity in AxSpA has been reported relatively infrequently, but it paradoxically appears to have an opposite effect compared to AS, i.e. HLA-B27 negative subjects with AxSpA generally report higher BASDAI scores than HLA-B27 positive subjects^16^. The reasons for this are not completely understood.

All classification or diagnostic criteria must achieve a balance between sensitivity and specificity. HLA-B27 fulfills a prominent role in the ASAS criteria for nr-AxSpA since only 3 criteria are required to make a diagnosis, and HLA-B27 can be one of the three^11^. While the presence of HLA-B27 is a useful discriminator, it also has intrinsic limitations with regard to sensitivity and specificity. For example, about 25% of patients with nr-AxSpA are HLA-B27 negative^17^.

We recently conducted a survey on spondyloarthritis and Covid-19^18,19^. This report analyzes the effect of HLA-B27 on the BASDAI scores in this cohort.

## Materials and methods

We have reported details about our survey on spondyloarthritis and Covid-19 elsewhere^18,19^.

Subjects were asked questions that included a modified BASDAI. The original BASDAI is based on six questions, the last two of which are both on morning stiffness. We relied on a single question on morning stiffness in order to shorten the survey and improve completion rates. In addition, because this was a web-based survey, BASDAI scores were reported as integers, not as a continuous variable. The study was reviewed and approved by the Oregon Health & Science University IRB, study number 0021375. As an electronic survey, subjects did not provide written informed consent, but they were explicitly informed that participating in the study was a form of giving consent. The research was performed in accordance with all relevant guidelines and regulations. The research complied with the recommendations made in the Declaration of Helsinki.

The chi-square test was used to compare frequencies of HLA-B27 among subjects with varying BASDAI scores or with variable use of a biologic. Linear regression was used to determine if specific variables such as treatment, HLA-B27 status, or sex could account for differences in the BASDAI score.

## Results

As recently reported (18), we recruited 3435 respondents with a median age of 52 from 65 countries, 74.5% from the United States and 8% were from Canada. Of the respondents who stated that they have physician-diagnosed spondyloarthritis, 2836 or 82.6% knew their HLA-B27 status and 76.1% were positive. 63.7% of those answering the survey were women. As previously reported^19^, 86% of respondents reported their disease as ankylosing spondylitis and 6.8% identified themselves as having nrAxSpA.

In analyzing results from this survey, we noted that HLA-B27 status had a statistically significant negative effect on the modified BASDAI (Table 1) (average BASDAI of 4.92 for the B27 negative group and 4.34 for the B27 positive group, p ≤ 0.0001 by chi square). Individual BASDAI questions each produced a similar difference between HLA-B27 positive and negative subjects. Women had an average BASDAI of 4.79 and men had an average BASDAI of 3.95. Women made up 73.9% of the HLA-B27 negative group and 60.5% of the HLA-B27 positive group. We used linear regression as shown in Table 2 to determine if sex, HLA-B27 status or use of a biologic independently affected the BASDAI. Both sex and HLA-B27 status do have independent effects on the BASDAI, while the score was not different in those taking a biologic compared to those not on a biologic.

**Table 1:** BASDAI score Relative to HLA-B27 status.

	HLA-B27 negative (%)	HLA-B27 positive (%)
BASDAI 1	4.9	10.5
BASDAI 2	9.9	15.8
BASDAI 3	13.7	14.3
BASDAI 4	15.0	13.5
BASDAI 5	14.4	14.0
BASDAI 6	17.5	12.4
BASDAI 7	13.0	10.3
BASDAI 8	7.7	6.2
BASDAI 9	2.7	2.2
BASDAI 10	1.3	0.9

This table shows the proportions of subjects with each BASDAI score with subjects analyzed by HLA-B27 status. The difference in BASDAI relative to B27 status is highly significant by Pearson’s chi-squared test, p < 0.0001.

**Table 2:** Linear regression analysis of the impact of sex, HLA-B27 positivity, or use of a Biologic on the BASDAI score.

	Regression coefficient estimate	Standard error	p-value
Intercept^a^	5.12	0.101	< 0.001
B27+	− 0.48	0.096	< 0.001
Male	− 0.79	0.085	< 0.001
Biologic (yes)	− 0.0001	0.053	0.999

^a^The intercept is the average BASDAI score for a female who is HLA-B27 negative and not taking a biologic. The regression coefficient shows how much lower the BASDAI would be on average if the respondent is HLA-B27 positive or male or taking a biologic.

Table 1 shows that HLA-B27 positive subjects were more than twice as likely to have a BASDAI of 1 and about 60% more likely to have a BASDAI of 2 compared HLA-B27 negative subjects (p ≤ 10^–4^ by two by two Chi square). Since this analysis included subjects with multiple different forms of spondylitis, we repeated the analysis but restricted the comparison just to the subset of subjects with self-described ankylosing spondylitis. Results were similar with HLA-B27 positive respondents 2.5 times as likely to have a BASDAI score of one and 65% more likely to have a BASDAI score of 2. A potential explanation is that HLA-B27 positive subjects are treated more aggressively than B27 negative subjects. Details on therapy for subjects in this cohort have been published previously^18^. Rather than finding that HLA-B27 positive subjects were treated more aggressively, we found the opposite, i.e. that 1736 of the 2836 subjects with known HLA type were taking a biologic (either anti-TNF or anti-IL-17), including 65.5% of those who were B27 negative on a biologic versus 59.9% of those who were B27 positive (p = 0.009 that HLA-B27 positive subjects were less likely to be on a biologic). A linear regression analysis (Table 2) failed to show that taking a biologic influenced the BASDAI, but the BASDAI scores represent disease activity with treatment; we do not have access to scores prior to treatment.

## Discussion

The finding of a negative effect of HLA-B27 on the activity of spondyloarthritis was the opposite of the results from at least 7 studies that have considered the effect of HLA-B27 on the BASDAI in patients with AS^3–9^. We are aware of only one study which found that HLA B27 positive subjects with AS had less active disease^20^. In contrast to the preponderance of data on AS, a single study on the effect of HLA-B27 on the activity of AxSpA concluded, as supported by our data, that HLA-B27 was associated with less disease activity^16^. A potential explanation for this paradox is that AS is more common in men, while AxSpA is more common in women^15,21^. Women have been shown to have higher BASDAI scores than men^15,21^. Further, the survey nature of our study likely contributes by including more women than men since women are known to participate more frequently in surveys compared to men^22^.

Sex differences, however, do not account fully for the difference which we found between HLA-B27 positive and negative subjects (see Table 2). Most studies on the effect of HLA-B27 on disease activity were conducted before the recognition of AxSpA using the ASAS criteria^3–8^. HLA-B27 positivity greatly facilitates the diagnosis of AxSpA by the ASAS criteria. We hypothesize that the ASAS criteria have encouraged clinicians to feel more comfortable diagnosing spondyloarthritis in an individual who is HLA-B27 positive. Thus, a patient who is HLA-B27 positive with mild back pain might be labelled AxSpA, while a subject with similar symptoms and a negative test for HLA-B27 is more likely to receive an alternative diagnosis such as fibromyalgia or mechanical low back pain, a possible example of a cognitive bias in diagnostic reasoning^23^ and subsequent misclassification of subjects.

We did not anticipate that patients who are HLA-B27 positive would report lower BASDAI scores than patients who are HLA-B27 negative. Physicians, however, are relatively intolerant of uncertainty^24^. A positive test for HLA-B27 could bias the diagnostic process toward attributing back pain to spondyloarthritis. Conversely, a negative test for HLA-B27 could unconsciously bias a clinician against making a diagnosis of spondyloarthritis for a patient with mild, chronic back pain.

Our data do not distinguish between over diagnosing AxSpA in a patient who is HLA-B27 positive versus under diagnosing the condition in someone who is HLA-B27 negative. Indeed, both are likely to be true.

This study has limitations which are inherent in data obtained by a survey. Most importantly, we believe that this is a study that relates to AxSpA rather than AS despite the fact that only 6.8% of respondents self-identified themselves as having nr-AxSpA, while 86% of respondents self-reported their disease as ankylosing spondylitis. All reported diagnoses related to spondyloarthritis in this survey have been previously reported^19^.

Based on our clinical experience with patients’ description of their own diagnosis, we question if respondents reliably distinguished AxSpA or nr-AxSpA from AS. Epidemiologic data indicate that AxSpA is common^15^. Further, subjects were allowed to provide more than one diagnosis such as acute anterior uveitis and AS. Since AS is a subset of AxSpA, more subjects should have had AxSpA than AS. In fact, an important observation from our study is that the term, axial spondyloarthritis, is apparently not commonly recognized by the lay public. Our conclusion that this is a study on spondyloarthritis and not AS is also supported by the predominance of female respondents and a lower prevalence of HLA-B27 than would be expected in a study of just ankylosing spondylitis.

Another limitation is that HLA-B27 status and diagnosis are by self-report and not independently validated. This weakness, however, is also a potential strength because the survey nature of the study allows us to capture subjects with mild disease and it provides insight into community practice. Community standards for diagnosing AxSpA are more broadly applicable than standards which might be used in a study based on subjects being evaluated at an academic medical center. Additional limitations include that the BASDAI relies on a subjective assessment and not on objective observations; and clearly, respondents to a survey represent a self-selected group. Despite these limitations, we believe that the observation is both provocative and heuristic. It is consistent with a prior report^16^ which did not attempt to explain why HLA-B27 should be associated with lower disease activity in AxSpA. It makes intuitive sense that a diagnostician would weigh the result of HLA-B27 testing excessively in assessing mild back pain. An acknowledgement of cognitive bias can lead to improved diagnostic accuracy and will encourage refinement of valuable guidelines^11^.

In summary, we are aware of only one other published study on the effect of HLA-B27 on the BASDAI score of patients with AxSpA^16^. Paradoxically, HLA-B27 is associated with lower disease activity in AxSpA and higher disease activity in AS. Sex differences contribute to this dichotomy, but they do not explain it completely. Treatment differences are also not an adequate explanation. Our observations from this unique survey population suggest that AxSpA is diagnosed disproportionately in HLA B27 positive subjects with mild back pain. This finding should be further validated by epidemiologic studies that do not rely on a survey instrument. And finally, the findings strongly suggest that ankylosing spondylitis is a term that is recognized far more frequently than axial spondyloarthritis by the lay public.

## Data Availability

Primary data from the survey may be obtained by contacting the corresponding author.
